# Identification of ceRNA Regulatory Networks Driven by the lncRNA NEAT1 in Multiple Myeloma

**DOI:** 10.1111/jcmm.71123

**Published:** 2026-04-02

**Authors:** Domenica Ronchetti, Valentina Traini, Ilaria Silvestris, Giuseppina Fabbiano, Andrea Devecchi, Federica Torricelli, Noemi Puccio, Ilaria Craparotta, Marco Bolis, Roberto Piva, Antonino Neri, Luca Agnelli, Francesco Passamonti, Niccolò Bolli, Elisa Taiana

**Affiliations:** ^1^ Department of Oncology and Hemato‐Oncology University of Milan Milan Italy; ^2^ Hematology Unit Fondazione IRCCS Ca' Granda Ospedale Maggiore Policlinico Milan Italy; ^3^ Department of Diagnostic Innovation Fondazione IRCCS Istituto Nazionale dei Tumori Milan Italy; ^4^ Laboratory of Translational Research Azienda USL‐IRCCS di Reggio Emilia Reggio Emilia Italy; ^5^ Computational Oncology Unit, Experimental Oncology Department Mario Negri IRCCS Milan Italy; ^6^ Bioinformatics Core Unit Institute of Oncology Research (IOR) Bellinzona Switzerland; ^7^ Department of Molecular Biotechnology and Health Sciences University of Turin Turin Italy; ^8^ Scientific Directorate Azienda USL‐IRCCS di Reggio Emilia Reggio Emilia Italy; ^9^ Department of Medical Oncology Fondazione IRCCS Istituto Nazionale Tumori Milan Italy

**Keywords:** ceRNA network, multiple myeloma, NEAT1

## Abstract

The lncRNA NEAT1 is overexpressed in multiple myeloma (MM) plasma cells and plays a key role in MM pathogenesis. NEAT1 is involved in ceRNA network in several cancers; however, data in MM are virtually absent. This study identified a NEAT1‐driven ceRNA network involving 96 miRNAs and 40 target genes, selected as concurrently downregulated in NEAT1‐KD AMO1 cells and upregulated in NEAT1‐overexpressing AMO1 cells (AMO1‐OVX). The co‐expression of NEAT1 and the targets was validated in MM patients (GSE116294, GSE13591, GSE6477, CoMMpass), and in NEAT1‐KD NCI‐H929, LP1, and KMS27 cell lines, showing for all targets a consistent downregulation, resembling that of NEAT1. The functional implication of the ceRNA network was explored by functional enrichment analyses of the 40 targets, identifying 78 significant gene sets, 17 of which were found significantly enriched by GSEA analysis in at least one experimental condition among NEAT1‐KD LP1, NCI‐H929, and KMS27 cells, AMO1‐OVX cells, or the extreme quartiles of NEAT1 expression in the CoMMpass dataset. Noteworthy, the cell cycle gene set was validated in 5 out of 6 conditions tested, suggesting that in MM the impact of NEAT1 upregulation on the cell cycle, experimentally demonstrated in our earlier publications, may be attributable, at least partially, to ceRNA mechanisms.

## 1 Introduction

Multiple myeloma (MM) is a malignancy of bone marrow plasma cells (PCs) with a different clinical course and characterised by a highly heterogeneous genetic background with both structural chromosomal alterations and specific gene mutations [1].

Long non‐coding RNAs (lncRNAs) form a group of non‐protein coding RNAs longer than 200 nucleotides, representing more than half of the mammalian non‐coding transcriptome [2]. LncRNAs are involved in many biological processes, such as cell proliferation, apoptosis, cellular differentiation, tumorigenesis and metastasis [2, 3]. LncRNAs act as essential components of complex gene regulatory network by modulating gene expression at the transcriptional, post‐transcriptional, and epigenetic levels [4]. In addition, lncRNAs play a key role in the regulation of gene expression together with microRNAs (miRNAs), which are small non‐coding RNAs of 19–25 nucleotides in length that can guide the post‐transcriptional repression of protein‐coding genes by binding to miRNA response elements (MREs) in the untranslated regions or the coding sequence of target genes [5]. Indeed, different RNA molecules harbouring MREs can compete for a common pool of miRNAs, thus acting as competing endogenous RNAs (ceRNAs), and this miRNA‐mediated circuits among transcripts can involve coding as well as noncoding transcripts, even lncRNAs [6]. It is now wide recognised that ceRNA network occurs widely in cellular processes, and that its perturbation will unsettle the transcriptomic equilibrium, potentially contributing to the development of pathological condition, including cancer [7]. In MM distinct lncRNAs transcriptional signatures distinguish MM PCs from their normal counterparts [8]. Among the differentially and most expressed lncRNAs, we identified the nuclear paraspeckle assembly transcript 1 (NEAT1) [9], already reported to be over‐expressed in many types of solid tumours, raising the hypothesis that it may play a critical oncogenic activity and facilitate tumorigenesis [10]. Accordingly, we previously demonstrated that NEAT1 silencing negatively regulates proliferation and viability of MM cells, both in vitro and in vivo [11]. NEAT1 represents the core structural component of the nuclear paraspeckles (PSs), which are lncRNA‐directed membraneless organelles involved in many different biological processes, including gene expression. Indeed NEAT1 regulates transcription and pre‐mRNA splicing events and holds nuclear mRNA for editing [12]. In MM, aberrant NEAT1 expression has been proven to be crucial for PCs survival under stressful conditions such as serum starvation and hypoxia. This effect is mediated through its regulation of PSs function, which supports the maintenance of DNA integrity [13]. In addition to its role within PS, a broad involvement of NEAT1 in different ceRNA mechanisms has been described in several different tumours [14]; nevertheless, data regarding NEAT1‐dependent ceRNA circuits in MM are very few and focused on specific NEAT1/miRNA/transcript axes [15, 16, 17, 18]. However, the possibility that a single transcript can be targeted by multiple different miRNAs, and that a single miRNA can regulate numerous transcripts, suggests that NEAT1 may affect pervasively post‐transcriptional regulation through broad, interconnected networks, rather than through isolated ceRNA pair interactions. These considerations open an interesting scenario on how NEAT1 overexpression in MM might broadly influence the expression of genes involved in tumour development and progression.

By analysing gene expression data from human MM cell lines (HMCLs) and large MM patient cohorts, this study revealed a novel NEAT1‐driven ceRNA network that may play a role in the pathogenesis of MM.

## 2 Materials and Methods

### 2.1 Multiple Myeloma Cell Lines

AMO1, NCI‐H929, and LP1 were purchased from DSMZ. Engineered AMO1^SAM^ gSCR and AMO1^SAM^ gN#8 (AMO1‐OVX) cell lines were obtained as previously described [13]. Details are reported in the Supporting Information.

### 2.2 Multi‐Omics Data in CoMMpass Study

Multi‐omics data about bone marrow MM samples at baseline were publicly accessible from MMRF CoMMpass Study (https://research.themmrf.org/) and retrieved from the Interim Analysis 20 (MMRF_CoMMpass_IA20, accessed on 19 January 2023). Details are reported in the Supporting Information.

### 2.3 RNA‐Sequencing

Libraries with optimal quality and quantity criteria were run on NextSeq 500 sequencer (Illumina). For gene expression analysis, we applied the voom/limma pipeline. Details are reported in the Supporting Information.

### 2.4 Survival Analysis

Survival analyses were performed using survival and survminer packages in R Bio‐conductor (version 4.1.2). The median cut‐point value was used to stratify MM cases of CoMMpass cohort into high and low target expression groups.

### 2.5 Statistical Analysis

Wilcoxon rank‐sum and Kruskal‐Wallis tests were applied to assess differential expression patterns between groups. Dunn's test was used for pairwise comparisons.

### 2.6 Enrichment Analysis

Gene set enrichment analysis (GSEA) was performed on the pre‐ranked differentially expressed protein‐coding genes based on the fold change, using Kegg, Reactome or Hallmark collections (v2024.1.Hs), by setting 15–500 gene set dimensions and performing 1000 permutations on samples (CoMMpass database) or gene sets (HMCLs), respectively. The Search Tool for the Retrieval of Interacting Genes (STRING) online tool (http://string‐db.org/) was applied to analyse the protein–protein interaction (PPI) of targets in the ceRNA network with the threshold of combined score > 0.40.

## 3 Results

### 3.1 Identification of Putative ceRNA Network Driven by NEAT1

To identify genes transcriptionally regulated by NEAT1, we performed RNA‐sequencing (RNA‐seq) analyses on AMO1 cell line under two opposing conditions previously optimised in our laboratory: NEAT1 knock‐down (KD) using a specific LNA‐gapmeR [11] and NEAT1 overexpression (NEAT1‐OVX), achieved through the CRISPR‐Cas9 SAM gain‐of‐function approach [13].

Specifically, the comparison of RNA‐seq data from NEAT1‐KD AMO1 cells with the relative control condition (SCR^gapmer^) revealed 515 differentially expressed genes (108 upregulated, 407 downregulated, FDR < 0.05). Similarly, the analysis of NEAT1‐OVX AMO1 cells relative to their control (SCR^CAS9^) identified 1256 deregulated genes (641 upregulated, 615 downregulated, FDR < 0.05).

**FIGURE 1:**
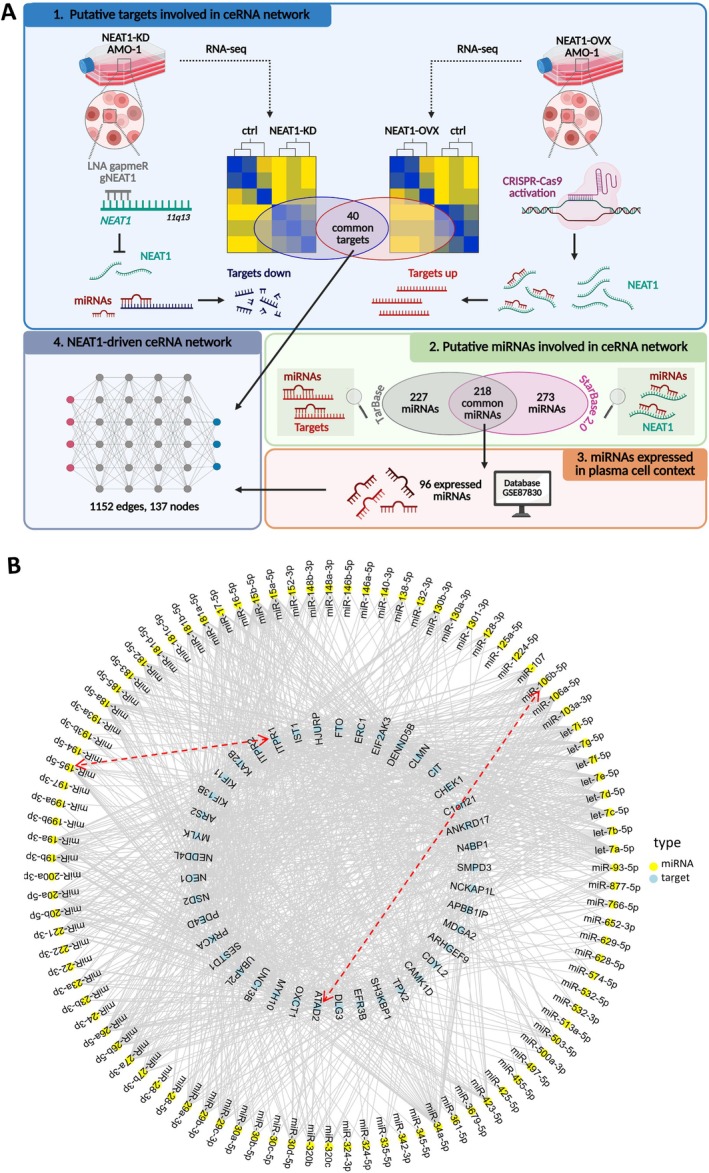
NEAT1‐driven ceRNA network. (A) Flowchart of analyses leading to the definition of NEAT1‐driven ceRNA network. (B) NEAT1‐driven ceRNA network of 96 miRNAs (yellow) and 40 targets (light blue). For better readability of the networks, NEAT1 node and edges with miRNAs are not represented. Red arrows indicate the 2 NEAT1‐driven ceRNA axes described in literature [19, 20].

To identify genes potentially involved in the NEAT1‐driven ceRNA network, we focused on those that were simultaneously downregulated in NEAT1‐KD and upregulated in NEAT1‐OVX AMO1 cells. Applying these criteria, we defined a list of 40 candidate mRNAs (see scheme in Figure 1A).

Afterwards, to identify miRNAs able to target these 40 candidate mRNAs, we interrogated the TarBase database [21] that collects experimentally supported miRNA–gene interactions. This analysis revealed a list of 445 miRNAs that have been shown to target the mRNAs identified as deregulated upon NEAT1 silencing or overexpression. Concurrently, to highlight putative miRNAs involved in the ceRNA network with NEAT1, we queried the StarBase 2.0 database [22], which can provide comprehensive predictions and experimental validations of lncRNA‐miRNA interactions. This analysis identified 491 miRNAs predicted and validated to bind NEAT1. By comparing this list with the 445 miRNAs targeting the 40 candidate mRNAs, we identified 218 miRNAs able to interact with both NEAT1 and at least one of the selected mRNAs, making them reasonable candidates for involvement in a NEAT1‐driven ceRNA network.

Based on this *in silico*‐derived list, we aimed to identify miRNAs with a potential functional role in MM by examining their expression levels in the cellular context of interest, that is, normal and pathological PCs. To this end, we analysed a proprietary database including CD138+ purified PCs samples derived from 4 healthy donors, 113 MM, and 30 plasma cell leukaemia (PCL) patients, profiled onto Affymetrix GeneChip miRNA 3.0 Arrays (GSE87830). Among the 218 candidate miRNAs, 96 exhibited appreciable expression levels within the PC context (log2 > 4 in at least 10 samples). Consequently, these 96 miRNAs were identified as having the potential to target the 40 transcripts deregulated by NEAT1 KD or overexpression (Figure 1 and Table S1), thus defining a potential ceRNA network composed of 1152 edges and 137 nodes (Figure 1B).

### 3.2 Expression and Clinical Impact of the NEAT1‐Driven ceRNA Network Targets in MM

To explore the relevance of the 40 target genes identified in the NEAT1‐driven ceRNA network in MM, we investigated their expression profiles in pathological PCs compared to normal controls. For this analysis, we used a proprietary dataset generated from GeneChip Affymetrix 2.0 arrays, including CD138+ purified PCs samples derived from 4 healthy donors (N) and 50 MM patients (GSE116294) [23]. We found that 7 out of 40 target genes are significantly upregulated in MM PCs compared to normal samples (Table 1).

**TABLE 1:** Summary of the characteristics of the 40 targets in MM. Table 1.

Gene name	Gene stable ID	Gene description	Karyotype band	UP‐REGULATION in MM vs. N^a^ (GSE116294)	OS^b^	PFS^b^
CLMN	ENSG00000165959	Calmin	14q32.13	*p* = 0.0390	—	—
UBAP2L	ENSG00000143569	Ubiquitin associated protein 2 like	1q21.3	*p* = 0.0305	*p* < 0.0001	*p* = 0.0012
NEO1	ENSG00000067141	Neogenin 1	15q24.1	*p* = 0.0280	—	—
PRKCA	ENSG00000154229	Protein kinase C alpha	17q24.2	*p* = 0.0199	—	—
ARHGEF9	ENSG00000131089	Cdc42 guanine nucleotide exchange factor 9	Xq11.2	*p* = 0.0166	—	—
DLG3	ENSG00000082458	Discs large MAGUK scaffold protein 3	Xq13.1	*p* = 0.0095	*p* = 0.0032	—
UNC13B	ENSG00000198722	Unc‐13 homologue B	9p13.3	*p* = 0.0031	—	—
MYLK	ENSG00000065534	Myosin light chain kinase	3q21.1	—	*p* = 0.029	*p* = 0.016
OXCT1	ENSG00000083720	3‐oxoacid CoA‐transferase 1	5p13.1	—	*p* = 0.017	*p* = 0.0009
IST1	ENSG00000182149	IST1 factor associated with ESCRT‐III	16q22.2	—	*p* = 0.016	—
C1orf21	ENSG00000116667	Chromosome 1 open reading frame 21	1q25.3	—	*p* = 0.014	*p* = 0.0035
FTO	ENSG00000140718	FTO alpha‐ketoglutarate dependent dioxygenase	16q12.2	—	*p* = 0.013	*p* = 0.023
SESTD1	ENSG00000187231	SEC14 and spectrin domain containing 1	2q31.2	—	*p* = 0.0057	*p* = 0.03
NSD2	ENSG00000109685	Nuclear receptor binding SET domain protein 2	4p16.3	—	*p* = 0.00095	*p* = 0.011
ATAD2	ENSG00000156802	ATPase family AAA domain containing 2	8q24.13	—	*p* < 0.0001	*p* < 0.0001
CHEK1	ENSG00000149554	Checkpoint kinase 1	11q24.2	—	*p* < 0.0001	*p* < 0.0001
CIT	ENSG00000122966	Citron rho‐interacting serine/threonine kinase	12q24.23	—	*p* < 0.0001	*p* < 0.0001
HJURP	ENSG00000123485	Holliday junction recognition protein	2q37.1	—	*p* < 0.0001	*p* < 0.0001
KIF11	ENSG00000138160	Kinesin family member 11	10q23.33	—	*p* < 0.0001	*p* < 0.0001
TPX2	ENSG00000088325	TPX2 microtubule nucleation factor	20q11.21	—	*p* < 0.0001	*p* < 0.0001
APBB1IP	ENSG00000077420	Amyloid beta precursor protein binding family B member 1 interacting protein	10p12.1	—	—	*p* = 0.011
ANKRD17	ENSG00000132466	Ankyrin repeat domain 17	4q13.3	—	—	—
CAMK1D	ENSG00000183049	Calcium/calmodulin dependent protein kinase ID	10p13	—	—	—
CDYL2	ENSG00000166446	Chromodomain Y like 2	16q23.2	—	—	—
DENND5B	ENSG00000170456	DENN domain containing 5B	12p11.21	—	—	—
EFR3B	ENSG00000084710	EFR3 homologue B	2p23.3	—	—	—
EIF2AK3	ENSG00000172071	Eukaryotic translation initiation factor 2 alpha kinase 3	2p11.2	—	—	—
ERC1	ENSG00000082805	ELKS/RAB6‐interacting/CAST family member 1	12p13.33	—	—	—
ITPR1	ENSG00000150995	Inositol 1,4,5‐trisphosphate receptor type 1	3p26.1	—	—	—
ITPR2	ENSG00000123104	Inositol 1,4,5‐trisphosphate receptor type 2	12p11.23	—	—	—
KAT2B	ENSG00000114166	Lysine acetyltransferase 2B	3p24.3	—	—	—
KIF13B	ENSG00000197892	Kinesin family member 13B	8p12	—	—	—
MDGA2	ENSG00000139915	MAM domain containing glycosylphosphatidylinositol anchor 2	14q21.3	—	—	—
MYH10	ENSG00000133026	Myosin heavy chain 10	17p13.1	—	—	—
N4BP1	ENSG00000102921	NEDD4 binding protein 1	16q12.1	—	—	—
NCKAP1L	ENSG00000123338	NCK associated protein 1 like	12q13.13	—	—	—
NEDD4L	ENSG00000049759	NEDD4 like E3 ubiquitin protein ligase	18q21.31	—	—	—
PDE4D	ENSG00000113448	Phosphodiesterase 4D	5q12.1	—	—	—
SH3KBP1	ENSG00000147010	SH3 domain containing kinase binding protein 1	Xp22.12	—	—	—
SMPD3	ENSG00000103056	Sphingomyelin phosphodiesterase 3	16q22.1	—	—	—

^a^ Significant targets upregulation in plasma cells purified from MM samples compared to those from healthy donors (N) is indicated by the corresponding *p*‐value (Wilcoxon test).

^b^ The significant clinical correlation of high target expression level with overall survival (OS) or progression free survival (PFS) is indicated by the corresponding *p*‐value (Log‐rank test *p*‐value).

Moreover, to assess the clinical relevance of the 40 target genes, we analysed transcriptomic and clinical data from 753 MM patients in the publicly available Multiple Myeloma Research Foundation (MMRF) CoMMpass database. Patients were stratified into high‐ and low‐expression groups for each gene, based on the median cut‐off value across the entire cohort.

This analysis revealed that elevated expression level of 13 target genes is significantly associated with poorer clinical outcome in terms of both overall survival (OS) and progression free survival (PFS). In addition, higher expression level of IST1 or DLG3 is associated with a significantly reduced OS but not PFS; conversely, higher APBB1IP expression level impacts PFS but not OS (Table 1).

### 3.3 Validation of the ceRNA Network in HMCLs and MM Patients Datasets

**FIGURE 2:**
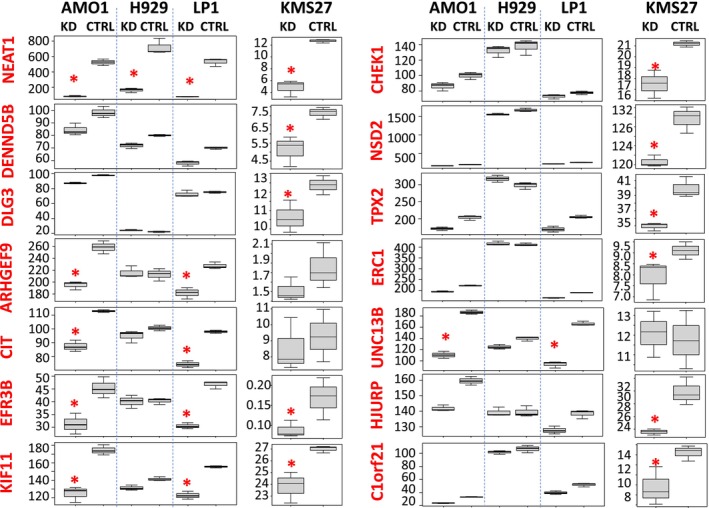
Boxplot of targets expression in NEAT1‐KD AMO1, NCI‐H929, LP1, and KMS27. Red asterisks indicate significant *p*‐value < 0.05.

To enhance confidence in the involvement of the 40 transcripts in the ceRNA network associated with NEAT1, we validated the modulation of the 40 targets upon NEAT1 silencing. Hence, we performed RNA‐seq analyses on NEAT1‐KD LP1 and NCI‐H929 cell lines, and we also analysed publicly available RNA‐seq data from NEAT1‐KD KMS27 cells (GSE178868). In nearly all analysed cell lines, the 40 transcripts displayed a consistent downregulation pattern similar to that of NEAT1, with 13 genes showing statistically significant modulation in at least one cell line (Figure 2, Figure S1).

Furthermore, we evaluated the positive correlation between NEAT1 and each of the 40 candidate mRNAs using gene expression data from three publicly available MM databases. The first was our previously mentioned proprietary dataset, including 50 MM patients (GSE116294) [23]. The second database comprises data from U133A Affymetrix arrays, where probes for 35/40 mRNAs are present, and includes purified CD138+ samples obtained from 217 MM patients (GSE13591 and GSE6477) [24, 25]. Lastly, we analysed expression profiles from 774 MM patients from the CoMMpass database. Across these datasets, 13 out of 40 candidate mRNAs showed a significantly positive Spearman's correlation with NEAT1 in at least one database. Notably, EIF2AK3, KAT2B, and NSD2 consistently exhibited significant correlation in two independent datasets (Table 2).

**TABLE 2:** Spearman's correlation analyses of 40 targets and NEAT1 expression levels in 3 MM patients databases.

Ensembl ID	Gene symbol	GSE13591 + GSE6477		GSE116294		CoMMpass	
		*R*	*p*	*R*	*p*	*R*	*p*
ENSG00000172071	**EIF2AK3**	**0.224**	**0.001**	**0.405**	**0.004**	−0.013	0.726
ENSG00000116667	**C1orf21**	−0.132	0.053	**0.299**	**0.035**	0.086	0.016
ENSG00000182149	**IST1**	0.120	0.079	**0.353**	**0.012**	0.063	0.078
ENSG00000114166	**KAT2B**	**0.315**	**0.000**	0.055	0.702	**0.196**	**0.000**
ENSG00000109685	**NSD2**	**0.223**	**0.001**	0.185	0.198	**0.131**	**0.000**
ENSG00000170456	**DENND5B**	**0.139**	**0.041**	−0.285	0.045	0.015	0.681
ENSG00000082458	**DLG3**	−0.186	0.006	0.148	0.303	**0.141**	**0.000**
ENSG00000140718	**FTO**	0.055	0.420	0.137	0.341	**0.106**	**0.003**
ENSG00000139915	**MDGA2**	—	—	0.049	0.736	**0.110**	**0.002**
ENSG00000133026	**MYH10**	−0.075	0.272	0.202	0.159	**0.150**	**0.000**
ENSG00000102921	**N4BP1**	−0.011	0.877	−0.076	0.597	**0.103**	**0.004**
ENSG00000123338	**NCKAP1L**	−0.095	0.161	0.003	0.984	**0.212**	**0.000**
ENSG00000147010	**SH3KBP1**	—	—	−0.090	0.533	**0.142**	**0.000**
ENSG00000132466	ANKRD17	0.052	0.443	−0.113	0.435	0.085	0.017
ENSG00000077420	APBB1IP	−0.112	0.101	−0.069	0.632	−0.002	0.946
ENSG00000131089	ARHGEF9	0.042	0.536	0.029	0.839	0.047	0.191
ENSG00000156802	ATAD2	−0.033	0.626	−0.126	0.383	−0.044	0.218
ENSG00000183049	CAMK1D	0.091	0.181	−0.212	0.140	0.083	0.021
ENSG00000166446	CDYL2	—	—	0.279	0.050	0.038	0.286
ENSG00000149554	CHEK1	—	—	−0.001	0.995	−0.066	0.068
ENSG00000122966	CIT	−0.061	0.368	−0.332	0.019	0.033	0.363
ENSG00000165959	CLMN	−0.182	0.007	−0.276	0.052	−0.046	0.201
ENSG00000084710	EFR3B	−0.210	0.002	0.247	0.084	0.084	0.019
ENSG00000082805	ERC1	−0.007	0.919	−0.309	0.029	−0.016	0.648
ENSG00000123485	HJURP	−0.153	0.024	−0.314	0.027	−0.045	0.213
ENSG00000150995	ITPR1	−0.082	0.231	−0.073	0.614	0.031	0.397
ENSG00000123104	ITPR2	−0.087	0.201	−0.303	0.033	0.071	0.047
ENSG00000138160	KIF11	−0.218	0.001	−0.313	0.027	−0.039	0.273
ENSG00000197892	KIF13B	−0.040	0.555	−0.040	0.781	0.034	0.351
ENSG00000065534	MYLK	−0.005	0.938	0.095	0.509	−0.021	0.562
ENSG00000049759	NEDD4L	0.109	0.110	−0.042	0.773	−0.037	0.307
ENSG00000067141	NEO1	0.059	0.390	−0.238	0.097	0.004	0.905
ENSG00000083720	OXCT1	0.070	0.306	−0.286	0.045	0.092	0.010
ENSG00000113448	PDE4D	−0.108	0.113	−0.201	0.160	−0.071	0.047
ENSG00000154229	PRKCA	−0.049	0.469	−0.187	0.193	−0.078	0.030
ENSG00000187231	SESTD1	—	—	0.201	0.160	0.039	0.282
ENSG00000103056	SMPD3	−0.058	0.394	−0.276	0.053	0.048	0.178
ENSG00000088325	TPX2	−0.044	0.518	−0.331	0.019	−0.044	0.223
ENSG00000143569	UBAP2L	0.000	0.998	0.176	0.221	0.072	0.046
ENSG00000198722	UNC13B	0.056	0.412	−0.436	0.002	0.073	0.044

*Note:* Targets with significant positive correlation are marked bold blue.

### 3.4 Genes Involved in the ceRNA Network Affects Mitotic Cell Cycle and Signal Transduction Processes

**FIGURE 3:**
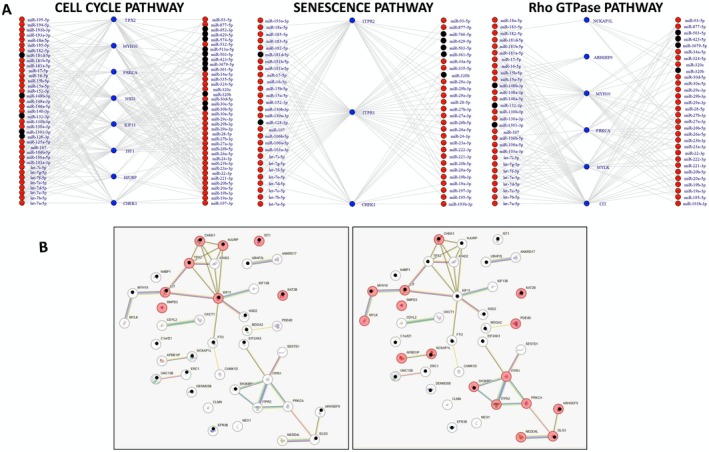
NEAT1‐driven ceRNA network affecting specific biological pathways. (A) ceRNA network involving targets enriched in the mitotic cell cycle pathway includes 84 nodes and 311 edges, in the senescence pathway includes 60 nodes and 154 edges, in the Rho GTPase pathway includes 66 nodes and 190 edges. For better readability of the networks, NEAT1 node and edges with miRNAs are not represented. Red bullets indicate miRNA relevant in MM reported in literature. (B) PPI network generated by STRING tool; pink coloured balls highlight proteins involved in cell cycle (left) and signal transduction (right). Black point in the balls indicates that the target has been validated in our analyses.

To explore the functional implication of the NEAT1‐driven ceRNA network, the 40 target mRNAs were subjected to functional enrichment analyses using EnrichR (https://maayanlab.cloud/Enrichr). Our analyses revealed that these genes are significantly enriched in 84 gene sets associated with several biological processes, including cell cycle, RHO GTPase signalling, and cell signalling (Table S2). To confirm that NEAT1 modulation could effectively affect these molecular pathways, we performed GSEA analyses on differentially expressed genes from NEAT1‐KD LP1, NCI‐H929, and KMS27 cells, AMO1‐OVX cells, and across the extreme quartiles of NEAT1 expression in the CoMMpass dataset. Seventeen gene sets resulted significantly enriched (NES ≤ −1.5 or NES ≥ 1.5, *p*_adj_ < 0.05) in at least one experimental condition. Notably, all validated pathways were significantly downregulated in samples with low NEAT1 expression levels or upregulated in association with high NEAT1 expression levels. To note, gene sets related to the cell cycle, cellular senescence, and RHO GTPase Effectors were consistently validated in 5 out of 6 experimental conditions (Table 3). Interestingly, focusing on the subnetworks including the targets enriched in these validated pathways, we identified a pool of miRNAs already known to be relevant in MM (Figure 3) [26, 27, 28].

**TABLE 3:** List of the Reactome, Kegg, and Hallmark gene sets found significantly enriched by the EnrichR analysis of the 40 targets, which are also significantly enriched in GSEA analyses of NEAT1‐KD NCI‐H929, LP1 and KMS27 cells, AMO1‐OVX cells, or in the extreme quartiles of NEAT1 expression in the CoMMpass dataset.

Pathways	AMO1 NEAT1‐KD		H929 NEAT1‐KD		LP1 NEAT1‐KD		KMS27 NEAT1‐KD		AMO1 NEAT1 OVX		CoMMpass	
	NES	FDR	NES	FDR	NES	FDR	NES	FDR	NES	FDR	NES	FDR
RHO GTPase effectors	−1.80	0.01			−1.92	0.00	−2.78	0.00	3.35	0.00	2.19	0.02
Cell cycle	−2.15	0.01			−1.91	0.00	−2.20	0.00	2.78	0.00	2.05	0.03
Cellular senescence	−1.66	0.03			−2.16	0.00	−2.06	0.01	3.34	0.00	2.06	0.02
VEGFR2 mediated cell proliferation	−1.96	0.03			−1.92	0.03						
Opioid signalling	−2.14	0.01			−2.14	0.00						
DAG and IP3 signalling	−1.91	0.02			−1.96	0.01						
Tight junction	−1.88	0.03			−1.73	0.04						
Regulation of actin cytoskeleton	−1.71	0.03			−1.59	0.05						
Mitotic spindle	−1.99	0.00			−1.55	0.01			2.36	0.00	2.46	0.00
E2F targets	−2.40	0.00			−2.13	0.00			2.08	0.01		
Haemostasis									2.01	0.00	1.71	0.10
Signalling by VEGF											1.82	0.06
Transmission across chemical synapses					−1.55	0.04						
Vascular smooth muscle contraction					−1.70	0.05						
Calcium signalling pathway					−1.72	0.04						
Axon guidance					−1.74	0.04					2.03	0.03
Signalling by receptor tyrosine kinases											1.87	0.05

Abbreviations: FDR = false discovery rate, NES = normalised enriched score.

To gain further insight into the functional impact of NEAT1‐mediated regulatory circuits, the STRING tool was applied to analyse the protein–protein interactions (PPI) among the 40 targets. The analysis identified a significant PPI network including 40 nodes and 33 edges (enrichment *p*‐value = 3.33e‐08), indicating that the proteins are at least partially biologically connected as a group. Interestingly, functional enrichment analysis of this network indicated significant overrepresentation of proteins involved in the cell cycle and signal transduction pathways (FDR = 0.0077 and FDR = 0.022, respectively; Figure 3).

Notably, most of the targets enriched in the cell cycle and Rho GTPase signalling pathways are among those validated across our previous analyses (Figure 3, Table 2).

Finally, to further validate the involvement of NEAT1‐driven ceRNA network in the cell cycle pathway, we investigated whether a subset of miRNAs included in this subnetwork could influence the expression levels of some of their specific targets involved in the cell cycle pathway (Figure 3A). Specifically, we focused on miR‐195, miR‐106a‐5p, and miR‐106b‐5p (already reported as involved in NEAT1‐driven ceRNA network [19, 20, 29, 30]) and KIF11, NSD2, PRKCA, ATAD2, and TPX2 targets. We found that the transfection of each miRNA mimic appears to negatively affect the expression levels of all targets in AMO‐1 cells. In particular, miR‐195 mimic transfection leads to a significant downregulation of all targets but PRKCA; miR‐106a‐5p significantly downregulates NSD2 and TPX2, and miR‐106b‐5p significantly downregulates TPX2 (Figure S1).

## 4 Discussion

In the last decade, NEAT1 dysregulation in MM and its role in tumorigenesis has been investigated by our and other groups [11, 13, 31, 32, 33]. This study explores the involvement of NEAT1 in post‐transcriptional regulation through ceRNA network mechanisms, an area that remains largely unexplored in MM, with previous studies focusing on only a few specific NEAT1/miRNA/target transcript axes [15, 16, 17, 18]. In particular, we generated a NEAT1‐driven ceRNA network comprising 40 target transcripts and 96 miRNAs, most of which have already been demonstrated to be important in MM (Table S1).

Our analyses further revealed that 7 targets are upregulated in MM PCs compared to normal ones, and that higher expression levels of 16 targets are significantly associated with worse prognosis, suggesting their possible relevance in the pathology (Table 1). To note, among the 40 identified target genes, we found NSD2, which encodes a methyltransferase that is involved in the recurrent chromosomal translocation t(4;14). This translocation results in IgH enhancer‐driven overexpression of NSD2 in up to 20% of MM patients; the oncogenic functions of NSD2 in MM are related to its methyltransferase catalytic activity, which can drive extensive epigenomic reprogramming, contributing to disease progression [34, 35]. Our data highlighted a significant positive correlation between NSD2 and NEAT1 expression levels (Table 2), with elevated NSD2 expression levels also being linked to worse prognosis. However, in MM patients carrying t(4;14) translocation, NSD2 displayed an abnormal spiked expression, not accompanied by a corresponding increase in NEAT1 expression, as we previously reported [36]. This observation suggests that the NEAT1/miRNAs/NSD2 ceRNA regulatory mechanism may be ineffective in this particular subset of MM patients. Conversely, this ceRNA axis could be relevant in MM patients lacking the t(4;14) translocation, where higher NSD2 expression levels remain in any case associated with worse OS but not PFS (Figure S3).

Importantly, NSD2 has been reported to promote STAT3 methylation and activation, thereby enhancing downstream STAT3 signalling activity [37]. Since STAT3 directly regulates NEAT1 transcription by modulating its promoter activity [38], these findings suggest that NEAT1 may engage in ceRNA‐driven positive‐feedback loops that ultimately reinforce its own expression. Similarly, NEAT1 may also participate in additional ceRNA‐mediated positive feedback circuits involving the targets KAT2B and CHEK1, both of which can modulate the activity of p53, a transcription factor known to directly induce NEAT1 expression [38]. In particular, full transcriptional activity of p53 requires the coactivator KAT2B [39], which enhances p53‐dependent gene expression, whereas CHEK1 can modulate p53 function either by directly contributing to its phosphorylation [40] or by activating DNA‐PK within the DNA‐damage response signalling cascade [41]. Overall, these findings reveal an additional regulatory layer controlling NEAT1 transcription in MM and broaden our understanding of how NEAT1 integrates upstream signalling pathways with the regulation of key biological processes.

The NEAT1‐driven ceRNA network was constructed through an integrated approach combining experimental data and *in silico* analyses, and our findings were corroborated with different approaches. First, we queried publicly available databases, including only those miRNA–target interactions that had been experimentally validated; indeed, recent advances in techniques enabled the high‐throughput sequencing of immunoprecipitated RNAs after crosslinking (such as CLIP‐Seq, HITS‐CLIP, and PAR‐CLIP) providing a biochemical method to identify relevant miRNA‐target interactions [21, 22, 42]. Second, we selectively focused our analysis considering only miRNAs with appreciable expression levels within the PC context, thus ensuring biological relevance. Lastly, we validated the deregulation of selected targets that showed a positive correlation with NEAT1 expression in different HMCLs and across multiple large cohorts of MM patients.

Of note, two of the NEAT1/miRNA/target axes that we found in MM have already been demonstrated in other cellular contexts (Figure 2). Specifically, in papillary thyroid cancer, the overexpressed NEAT1 can function as a ceRNA to regulate the ATPase family AAA domain‐containing 2 (ATAD2) gene expression by sponging miR‐106b‐5p [19]. The second identified axis involves NEAT1/miR‐195‐5p/ITPR1, which has been described in colorectal cancer as part of a ceRNA network associated with immune infiltration [20].

Despite the importance of single ceRNA axes, the broader ceRNA network hypothesis suggests that NEAT1 may exert widespread influence on the post‐transcriptional regulation of tumour‐related gene expression. Interestingly, functional enrichment analyses of the target genes within the NEAT1‐driven ceRNA network revealed enrichment in key oncogenic pathways, especially those related to the cell cycle and RHO GTPase signalling. In particular, the targets enriched in the cell cycle pathway are IST1, CHEK1, TPX2, HJURP, PRKCA, and the previously discussed NSD2, highlighting the potential of NEAT1 to regulate multiple pivotal genes of cell proliferation and tumour progression. These targets have all proved to be relevant in MM; specifically, PRKCA is significantly upregulated in tumour PCs compared to healthy control, whereas the remaining five genes, IST1, CHEK1, TPX2, HJURP, and NSD2, could have clinical significance, as their high expression levels are significantly associated with a worse prognosis in MM patients (Table 1). Of note, TPX2 is an allosteric regulator of AURKA, and it is essential for correctly positioning AURKA at the mitotic spindle during cell division [43]. We recently reported that the expression of TPX2 in MM is associated with NEAT1 deregulation, leading to the hypothesis that NEAT1‐mediated perturbation of TPX2, involved in the control of AURKA activity, may contribute to mitotic instability in MM cells [31].

Along with TPX2, also for Cetron Rho‐interacting serine/threonine kinase (CIT) gene, which is involved in the RHO GTPase pathway (Table S2), the overexpression in MM in association with tumorigenesis has already been described [44]. CIT is a serine/threonine kinase crucial for cytokinesis, and it is upregulated in PCs of MM patients compared with healthy controls. Functional studies have shown that CIT silencing in HMCLs induces cytokinesis failure and reduces MM cell proliferation both in vitro and in vivo [44]. In the present study, we confirmed that the expression levels of both TPX2 and CIT are positively correlated with those of NEAT1, suggesting that NEAT1 overexpression in MM could imbalance the transcriptomic equilibrium through ceRNA mechanisms, resulting in the upregulation of target genes important for neoplastic proliferation. Furthermore, our experimental data indicate that the imbalances of specific miRNAs in the ceRNA network actually reduce the expression levels of genes involved in the cell cycle pathway, including KIF11, NSD2, PRKCA, ATAD2, and TPX2. It is not surprising that the downmodulation of some targets is less than 20%, considering that the NEAT1‐driven ceRNA network is characterised by a complex regulatory architecture, in which a single transcript may be targeted by multiple distinct miRNAs, and a single miRNA can modulate the expression of several transcripts. This intricate interplay suggests that NEAT1 exerts its influence on the post‐transcriptional regulation through broad, interconnected networks, rather than through isolated ceRNA pair interactions. Such complexity inherently limits the feasibility of validating individual components or isolated interactions within the ceRNA network.

In line with this hypothesis, our previous study experimentally demonstrated that NEAT1 silencing in MM cells impairs proliferation and viability, affects the cell cycle, and promotes apoptosis in vitro, ex vivo, and in vivo [11]. Taken together, these data suggest that the NEAT1‐driven ceRNA network might represent, at least in part, the molecular mechanism underlying these biological effects in MM.

## 5 Conclusion

This study strengthens the pivotal role of NEAT1 in MM. Furthermore, our results highlight NEAT1 as a master regulator of gene expression, operating at multiple levels. At the transcriptional level, NEAT1 has been reported to bind active chromatin sites, participating in transcriptional processes [45]. Moreover, through the assembling of PSs, NEAT1 acts as a molecular sponge for RNA binding proteins and PSs proteins. Although this sequestration does not impair their intrinsic functionality, it affects their interaction with native target genes, effectively resulting in a loss of function outcome. Importantly, both NEAT1 and PSs are upregulated in MM upon specific stress‐related conditions, such as hypoxia and serum deprivation, suggesting their involvement in modulating gene expression at the post‐translational level in response to microenvironmental stimuli [12]. Finally, our identification of a NEAT1‐driven ceRNA network further expands its regulatory influence to the post‐transcriptional level, offering additional insight into how NEAT1 may orchestrate gene expression changes that contribute to MM pathogenesis.

## Author Contributions

D.R., V.T., I.S., G.F., A.D., F.T., N.P., I.C. and E.T. performed experiments and analysed the data. M.B., R.P., L.A., A.N., F.P. and N.B. provided critical evaluation of experimental data and of the manuscript. D.R. and E.T. conceived the study and wrote the manuscript.

## Funding

This work was financially supported by grants from Associazione Italiana Ricerca sul Cancro (AIRC) to ET (MFAG 2022‐ID.27606) and to AN (IG 2020‐ID.24365). N.B. is funded by the European Research Council under the European Union's Horizon 2020 research and innovation programme (grant agreement no. 817997) and by Associazione Italiana Ricerca sul Cancro (IG25739). V.T. is supported by a fellowship from the PhD programme in Experimental Medicine of the University of Milan. The work was partially funded by the Italian Ministry of Health (current research IRCCS).

## Ethics Statement

All patients' data are derived from publicly available datasets.

## Conflicts of Interest

The authors declare no conflicts of interest.

## Supporting information


**Appendix S1:** jcmm71123‐sup‐0001‐Supinfo.pdf.

## Data Availability

RNAseq data from NEAT1‐KD AMO1, NCI‐H929, and LP1 have been deposited in the NCBI Gene Expression Omnibus database (GEO) and are accessible under accession #GSE304590. RNAseq data from NEAT1‐OVX AMO1 have been deposited at ArrayExpress; access code: E‐MTAB‐16772.
