# Supplementary material for: Identification of ceRNA Regulatory Networks Driven by the lncRNA NEAT1 in Multiple Myeloma
Source: J Cell Mol Med. 2026 Apr 2;30(7):e71123. doi: 10.1111/jcmm.71123 (PMC13051828; doi:10.1111/jcmm.71123)
Supplement: Supplementary file 1 — Appendix S1: jcmm71123‐sup‐0001‐Supinfo.pdf. [file JCMM-30-e71123-s001.pdf]

Supplementary data

Identification of ceRNA regulatory networks driven by the lncRNA NEAT1 in multiple myeloma

Authors:

Domenica Ronchetti,[1] Valentina Traini,[1] Ilaria Silvestris,[1] Giuseppina Fabbiano,[2] Andrea Devecchi,[3] Federica Torricelli,[4] Noemi Puccio,[4] Ilaria Craparotta,[5] Marco Bolis,[5,6] Roberto Piva,[7] Antonino Neri,[8] Luca Agnelli,[3,9] Francesco Passamonti,[1,2] Niccolò Bolli,[1,2] Elisa Taiana[2]

[1]Department of Oncology and Hemato-Oncology, University of Milan, Milan, Italy;

[2]Hematology Unit, Fondazione IRCCS Ca' Granda Ospedale Maggiore Policlinico, Milan, Italy;

[3]Department of Diagnostic Innovation, Fondazione IRCCS Istituto Nazionale dei Tumori, Milan, Italy;

[4]Laboratory of Translational Research, Azienda USL-IRCCS di Reggio Emilia, Reggio Emilia, Italy;

[5]Computational Oncology Unit, Experimental Oncology Department, Mario Negri IRCCS, Milan, Italy;

[6]Bioinformatics Core Unit, Institute of Oncology Research (IOR), Bellinzona, Switzerland;

[7]Department of Molecular Biotechnology and Health Sciences, University of Turin, Turin, Italy;

[8]Scientific Directorate, Azienda USL-IRCCS di Reggio Emilia, Reggio Emilia, Italy;

[9]Department of Medical Oncology, Fondazione IRCCS Istituto Nazionale Tumori, Milan, Italy

Supplementary methods

Multiple myeloma cell lines

Human multiple myeloma cell lines (HMCLs) AMO1, NCI-H929, and LP1 were purchased from DSMZ, which certified authentication performed by short tandem repeat DNA typing. All these cell lines were immediately expanded and frozen upon arrival and used from the original stock within 6 months. HMCLs were cultured in RPMI-1640 medium (Gibco®, Life Technologies, Carlsbad, CA, USA) supplemented with 10% fetal bovine serum, 1% penicillin-streptomycin (Euroclone, Milan, Italy) at 37°C in 5% CO_2 atmosphere. All HMCLs were routinely tested to exclude Mycoplasma contamination using Lonza Mycoalert Mycoplasma Detection Kit (Euroclone, Milan, Italy).

NEAT1 knock-down (KD) at 48h was obtained by gymnosis. Cells were seeded at low plating density (5×10^4 /ml) and treated with the naked gapmeRs g#N1_E [11] and the scrambled g#SCR, at the same time of the seeding at a final concentration of 5 μM.

RNA extraction

Total RNA was extracted using RNeasy kit (Qiagen) according to manufacturer's instructions. The purity and concentration of total RNA was determined by the NanoDrop 1000 spectrophotometer (Thermo Fisher Scientific). The ratios of absorption (260 nm/280 nm) of all samples were between 1.8 and 2.0.

Multi-Omics Data in CoMMpass Study

Multi-omics data about bone marrow MM samples at baseline (BM_1) were publicly accessible from MMRF CoMMpass Study (https://research.themmrf.org/) including more than 1000 MM patients from several worldwide sites and retrieved from the Interim Analysis 20 (MMRF_CoMMpass_IA20, accessed on 19 January 2023). Transcript per Million (TPM) reads values of the 40 target transcripts were retrieved using Salmon gene expression quantification data (MMRF_CoMMpass_IA20_salmon_geneUnstranded_TPM) in 767 BM_1 MM patients. Clinical data regarding Overall Survival (OS) and Progression free Survival (PFS) were considered in 753 MM patients for which both RNA-seq expression and survival data were available.

RNA-sequencing

Before library preparation, RNA concentration was evaluated through Qubit™ RNA Broad Range Assay Kit (Invitrogen, Walthman, MA, USA) while RNA quality was established on 4200 TapeStation (Agilent Technologies, Santa Clara, CA, USA) using RNA Screen tape kit (Agilent

Technologies, Santa Clara, CA, USA). According to the TruSeq Stranded Total RNA (San Diego, CA, USA) protocol, 500 ng of RNA for each sample with RIN value between 9 and 10, were used for RNA sequencing. Final libraries with optimal quality and quantity criteria, assessed by D1000 Screen tape kit (Agilent Technologies) and by Qubit® dsDNA High Sensitivity Assay Kit (Invitrogen), respectively, were run on NextSeq 500 sequencer (Illumina) using a 2 × 150 high-output flow cell with 8 samples/run. Raw sequencing data underwent quality assessment using FastQC. High-quality reads were aligned to the human reference genome (hg38) using the STAR aligner (v2.7.10a). Gene-level quantification was performed with featureCounts (v1.6.4) to obtain raw read counts. For gene expression analysis, we applied the voom/limma pipeline. Genes with fewer than 10 reads in more than 95% of samples were excluded to reduce background noise. Normalization was conducted using the trimmed mean of M-values (TMM) method to account for differences in library size and reduce technical variability. The data were then transformed using voom, which converts raw counts into log2 counts per million (log2-CPM) and assigns precision weights to each observation. Differential expression analysis was performed using these transformed values, and P values were adjusted for multiple comparisons using the Benjamini-Hochberg false discovery rate (FDR) method. Differential expressed genes were selected at 10% FDR adjusted p-value and 1.5 fold change cut off values.

miRNA-mimic transfection, reverse transcription and quantitative PCR

AMO-1 cells seeded at 200.000 cells/mL were transfected with miRNA mimics by Neon Transfection System (Invitrogen, CA, US), with the following electroporation conditions: 1100 V, 30 ms, 2 pulse. The miRNA mimics for has-miR-106b-5p, has- miR-106a-5p, and has-miR195-5p (Thermo Fisher Scientific: MC10067, MC10827, MC12567, respectively) were used at 100nM. 72h after transfection, total RNA was extracted using TRIzol® Reagent (Invitrogen, Life Technologies) according to the manufacturer's instructions. The purity and concentration of total RNA were determined by the NanoDrop 1000 spectrophotometer (Thermo Fisher Scientific). The ratios of absorption (260 nm/280 nm) of all samples were between 1.8 and 2.0. cDNA was synthesized from 500 ng of total RNA with random primers using the High Capacity cDNA Reverse Transcriptase Kit (Invitrogen) according to the manufacturer`s instructions. Real-time PCR was performed in triplicate using TaqMan® Assays for NSD2, KIF11, PRKCA, TPX2, and ATAD2 (Hs00983720_m1, Hs00189698_m1, and Hs00925200_m1, Hs00201616_m1, Hs00204205_m1, respectively) together with the TaqMan Universal PCR Master Mix on an Applied Biosystems 7900 Sequence Detection System. Data were analyzed using the $2^{-\Delta\Delta Ct}$ method to measure the relative changes in each gene's expression compared with negative control and GAPDH expression.

Supplementary Figure S1. Boxplot of target expression in NEAT1-KD AMO1, H929, LP1, and KMS27. For AMO1, NCI-H929, LP1 the target expression levels on the y-axis are expressed in log2-CPM, while for KMS27 in Transcript Per Million (TPM). Red asterisk indicated significant p-value <0.05

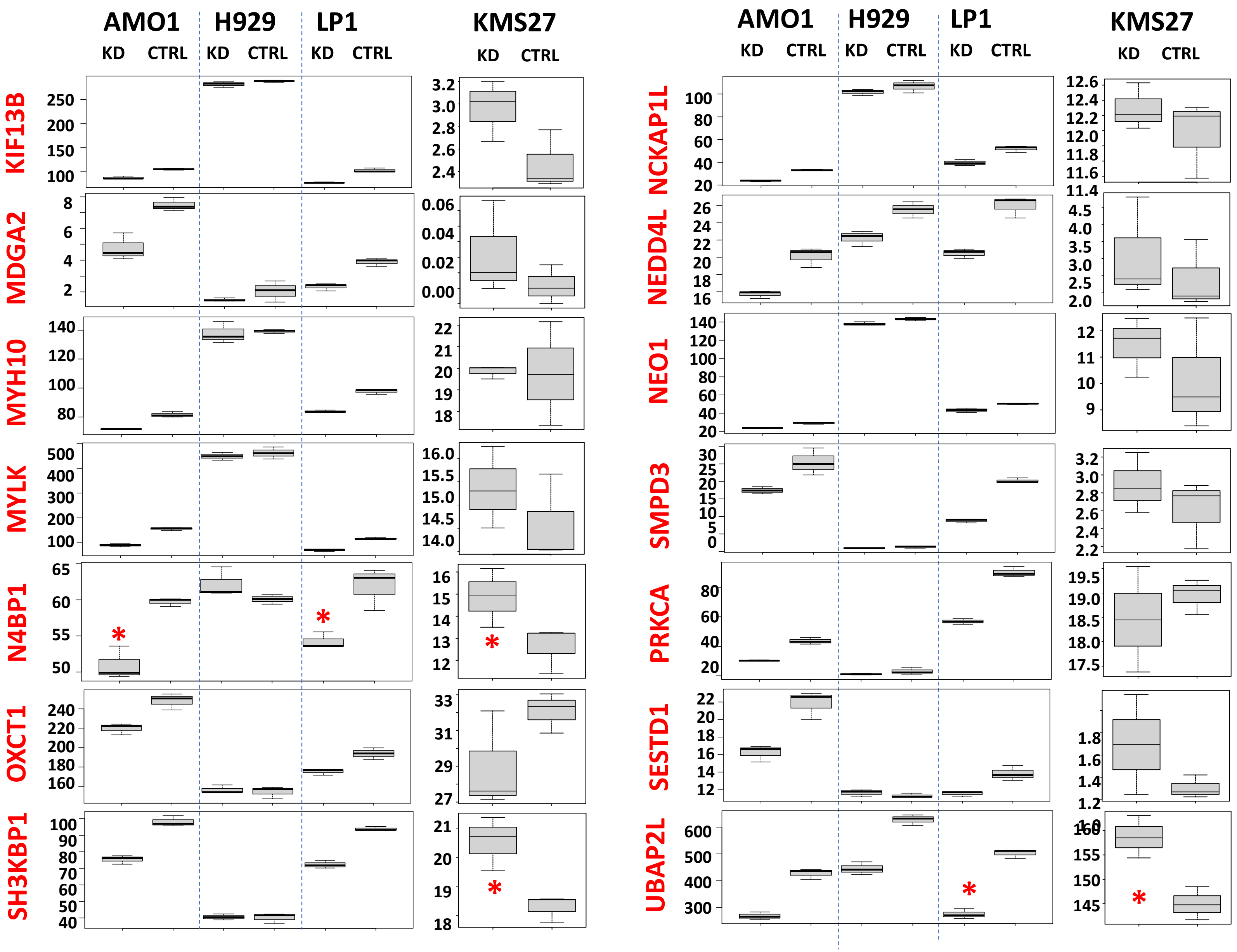

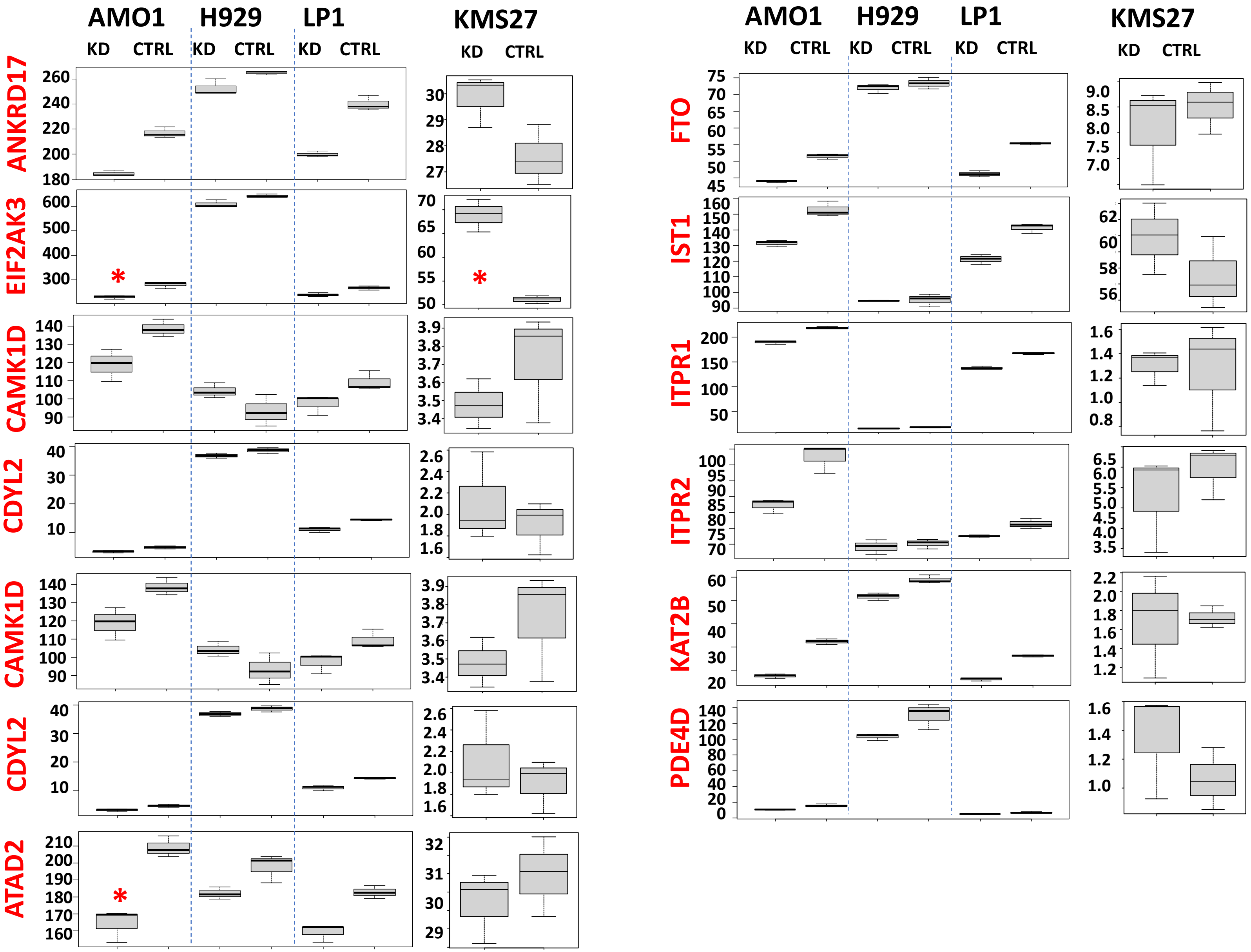
AMO1
H929
LP1
KMS27
KD
CTRL
ANKRD17
EIF2AK3
CAMK1D
CDYL2
CAMK1D
CDYL2
ATAD2
AMO1
H929
LP1
KMS27
KD
CTRL
FTO
IST1
ITPR1
ITPR2
KAT2B
PDE4D

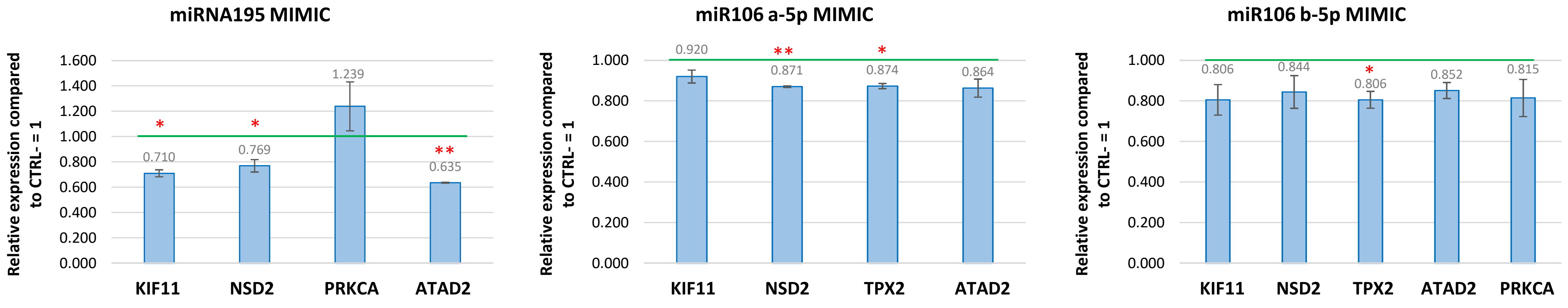

Supplementary Figure S2. Quantitative real-time PCR of target genes specific for each miRNA mimic in AMO-1 cells. * p value <0.05, ** p value <0.01

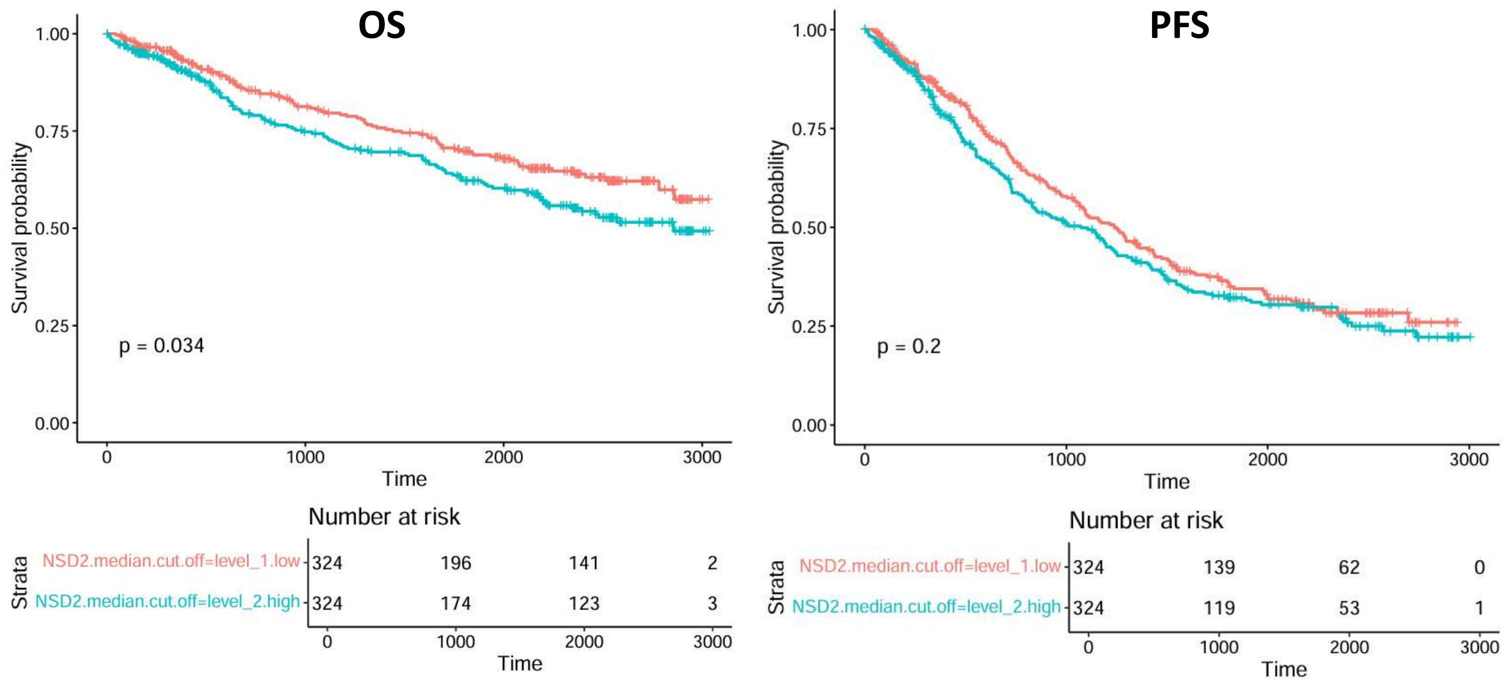

Supplementary Figure S3. Kaplan-Meier survival curves in the CoMMpass dataset including the subset of 648 MM not carrying t(4;14) chromosomal translocation. MM cases were stratified in high and low NSD2 expression groups, accordingly to the median expression level across the dataset. Log-rank test p-value measuring the global difference between survival curves and number of samples at risk in each group across time are reported

Supplementary Table S1: list of 40 genes and 96 miRNAs potentially involved in the NEAT1-driven ceRNA network; references of studies reporting their involvement in multiple myeloma are reported.

40 GENE LIST	Study in MM (REF)	96 miRNA GENE LIST		Study in MM (REF)
**ANKRD17**	-	**hsa-let-7a-5p**	MIMAT0000062	1
**APBB1IP**	-	**hsa-let-7b-5p**	MIMAT0000063	1
**ARHGEF9**	-	**hsa-let-7c-5p**	MIMAT0000064	1
**ATAD2**	-	**hsa-let-7d-5p**	MIMAT0000065	1
**C1orf21**	-	**hsa-let-7e-5p**	MIMAT0000066	1
**CAMK1D**	2	**hsa-let-7f-5p**	MIMAT0000067	1
**CDYL2**	-	**hsa-let-7g-5p**	MIMAT0000414	1
**CHEK1**	3,4	**hsa-let-7i-5p**	MIMAT0000415	1
**CIT**	5	**hsa-miR-103a-3p**	MIMAT0000101	1
**CLMN**	-	**hsa-miR-106a-5p**	MIMAT0000103	6
**DENND5B**	-	**hsa-miR-106b-5p**	MIMAT0000680	6
**DLG3**	-	**hsa-miR-107**	MIMAT0000104	1
**EFR3B**	-	**hsa-miR-1224-5p**	MIMAT0005458	-
**EIF2AK3**	7	**hsa-miR-125a-5p**	MIMAT0000443	8
**ERC1**	-	**hsa-miR-128-3p**	MIMAT0000424	-
**FTO**	9,10	**hsa-miR-1301-3p**	MIMAT0005797	-
**HJURP**	11	**hsa-miR-130a-3p**	MIMAT0000425	12
**IST1**	-	**hsa-miR-130b-3p**	MIMAT0000691	13
**ITPR1**	-	**hsa-miR-132-3p**	MIMAT0000426	-
**ITPR2**	-	**hsa-miR-138-5p**	MIMAT0000430	14,15
**KAT2B**	-	**hsa-miR-140-3p**	MIMAT0004597	16
**KIF11**	17,18	**hsa-miR-146a-5p**	MIMAT0000449	19
**KIF13B**	-	**hsa-miR-146b-5p**	MIMAT0002809	20
**MDGA2**	-	**hsa-miR-148a-3p**	MIMAT0000243	21
**MYH10**	22	**hsa-miR-148b-3p**	MIMAT0000759	-
**MYLK**	-	**hsa-miR-152-3p**	MIMAT0000438	23
**N4BP1**	-	**hsa-miR-15a-5p**	MIMAT0000068	1
**NCKAP1L**	-	**hsa-miR-15b-5p**	MIMAT0000417	24
**NEDD4L**	25,26	**hsa-miR-16-5p**	MIMAT0000069	1
**NEO1**	-	**hsa-miR-17-5p**	MIMAT0000070	6
**NSD2**	27–29	**hsa-miR-181a-5p**	MIMAT0000256	30
**OXCT1**	-	**hsa-miR-181b-5p**	MIMAT0000257	31
**PDE4D**	32	**hsa-miR-181c-5p**	MIMAT0000258	-
**PRKCA**	33,34	**hsa-miR-181d-5p**	MIMAT0002821	-
**SESTD1**	-	**hsa-miR-182-5p**	MIMAT0000259	35,36
**SH3KBP1**	-	**hsa-miR-183-5p**	MIMAT0000261	37
**SMPD3**	-	**hsa-miR-185-5p**	MIMAT0000455	38
**TPX2**	39,40	**hsa-miR-18a-5p**	MIMAT0000072	6
**UBAP2L**	-	**hsa-miR-18b-5p**	MIMAT0001412	-
**UNC13B**	-	**hsa-miR-193a-3p**	MIMAT0000459	41,42
		**hsa-miR-193b-3p**	MIMAT0002819	43
		**hsa-miR-194-5p**	MIMAT0000460	44

**hsa-miR-195-5p**	MIMAT0000461	45
**hsa-miR-197-3p**	MIMAT0000227	46–48
**hsa-miR-199a-3p**	MIMAT0000232	49
**hsa-miR-199b-3p**	MIMAT0004563	(49)
**hsa-miR-19a-3p**	MIMAT0000073	6
**hsa-miR-19b-3p**	MIMAT0000074	6
**hsa-miR-200c-3p**	MIMAT0000617	50
**hsa-miR-20a-5p**	MIMAT0000075	6
**hsa-miR-20b-5p**	MIMAT0001413	6
**hsa-miR-221-3p**	MIMAT0000278	51
**hsa-miR-222-3p**	MIMAT0000279	51
**hsa-miR-22-3p**	MIMAT0000077	52
**hsa-miR-23a-3p**	MIMAT0000078	53
**hsa-miR-23b-3p**	MIMAT0000418	54
**hsa-miR-24-3p**	MIMAT0000080	53
**hsa-miR-26a-5p**	MIMAT0000082	55
**hsa-miR-26b-5p**	MIMAT0000083	56
**hsa-miR-27a-3p**	MIMAT0000084	51
**hsa-miR-27b-3p**	MIMAT0000419	57
**hsa-miR-28-5p**	MIMAT0000085	58
**hsa-miR-28-3p**	MIMAT0004502	59
**hsa-miR-29a-3p**	MIMAT0000086	51
**hsa-miR-29b-3p**	MIMAT0000100	51
**hsa-miR-29c-3p**	MIMAT0000681	45
**hsa-miR-30a-5p**	MIMAT0000087	60
**hsa-miR-30b-5p**	MIMAT0000420	-
**hsa-miR-30c-5p**	MIMAT0000244	-
**hsa-miR-30d-5p**	MIMAT0000245	1
**hsa-miR-320c**	MIMAT0005793	61
**hsa-miR-320b**	MIMAT0005792	-
**hsa-miR-324-3p**	MIMAT0000762	-
**hsa-miR-324-5p**	MIMAT0000761	51
**hsa-miR-335-5p**	MIMAT0000765	62
**hsa-miR-342-3p**	MIMAT0000753	51
**hsa-miR-345-5p**	MIMAT0000772	-
**hsa-miR-34a-5p**	MIMAT0000255	1
**hsa-miR-361-5p**	MIMAT0000703	-
**hsa-miR-3679-5p**	MIMAT0018104	-
**hsa-miR-423-5p**	MIMAT0004748	-
**hsa-miR-425-5p**	MIMAT0003393	63
**hsa-miR-455-5p**	MIMAT0003150	-
**hsa-miR-497-5p**	MIMAT0002820	51
**hsa-miR-500a-3p**	MIMAT0002871	-
**hsa-miR-503-5p**	MIMAT0002874	-
**hsa-miR-513a-5p**	MIMAT0002877	-
**hsa-miR-532-5p**	MIMAT0002888	64,65
**hsa-miR-532-3p**	MIMAT0004780	64,65
**hsa-miR-574-5p**	MIMAT0004795	-
**hsa-miR-628-5p**	MIMAT0004809	-

**hsa-miR-629-5p**	MIMAT0004810	-
**hsa-miR-652-3p**	MIMAT0003322	-
**hsa-miR-766-5p**	MIMAT0022714	-
**hsa-miR-877-5p**	MIMAT0004949	66
**hsa-miR-93-5p**	MIMAT0000093	6

REFERENCES

1. Dubaj, M. *et al.* Role of Non-Coding RNAs in Diagnosis, Prediction and Prognosis of Multiple Myeloma. *Cancers* **16**, 1033 (2024).
2. Volpin, V. *et al.* CAMK1D Triggers Immune Resistance of Human Tumor Cells Refractory to Anti-PD-L1 Treatment. *Cancer Immunol. Res.* **8**, 1163–1179 (2020).
3. Zhou, L. *et al.* Chk1 Inhibition Potently Blocks STAT3 Tyrosine705 Phosphorylation, DNA-Binding Activity, and Activation of Downstream Targets in Human Multiple Myeloma Cells. *Mol. Cancer Res. MCR* **20**, 456–467 (2022).
4. Gu, C. *et al.* CHEK1 and circCHEK1_246aa evoke chromosomal instability and induce bone lesion formation in multiple myeloma. *Mol. Cancer* **20**, 84 (2021).
5. Sahin, I. *et al.* Citron Rho-interacting kinase silencing causes cytokinesis failure and reduces tumor growth in multiple myeloma. *Blood Adv.* **3**, 995–1002 (2019).
6. Feng, M. *et al.* Systematic analysis of berberine-induced signaling pathway between miRNA clusters and mRNAs and identification of mir-99a∼125b cluster function by seed-targeting inhibitors in multiple myeloma cells. *RNA Biol.* **12**, 82–91 (2015).
7. Dostálová, A. *et al.* Diff erent expression of genes involved in unfolded protein response in multiple myeloma and extramedullary dis ease patients. *Klin. Onkol. Cas. Ceske Slov. Onkol. Spolecnosti* **38**, 45–51 (2025).
8. Yu, H., Peng, S., Chen, X., Han, S. & Luo, J. Long non-coding RNA NEAT1 serves as a novel biomarker for treatment response and survival profiles via microRNA-125a in multiple myeloma. *J. Clin. Lab. Anal.* **34**, e23399 (2020).
9. Xu, A. *et al.* FTO promotes multiple myeloma progression by posttranscriptional activation of HSF1 in an m6A-YTHDF2-dependent manner. *Mol. Ther. J. Am. Soc. Gene Ther.* **30**, 1104–1118 (2022).
10. Wang, C., Li, L., Li, M., Wang, W. & Jiang, Z. FTO promotes Bortezomib resistance via m6A-dependent destabilization of SOD2 expression in multiple myeloma. *Cancer Gene Ther.* **30**, 622–628 (2023).
11. Jia, Y. *et al.* Super Enhancer-Mediated Upregulation of HJURP Promotes Growth and Survival of t(4;14)-Positive Multiple Myeloma. *Cancer Res.* **82**, 406–418 (2022).
12. Kubiczkova, L. *et al.* Circulating serum microRNAs as novel diagnostic and prognostic biomarkers for multiple myeloma and monoclonal gammopathy of undetermined significance. *Haematologica* **99**, 511–518 (2014).
13. Du, L. *et al.* SUMOylation inhibition enhances dexamethasone sensitivity in multiple myeloma. *J. Exp. Clin. Cancer Res. CR* **41**, 8 (2022).
14. Rastgoo, N., Pourabdollah, M., Abdi, J., Reece, D. & Chang, H. Dysregulation of EZH2/miR-138 axis contributes to drug resistance in multiple myeloma by downregulating RBPMS. *Leukemia* **32**, 2471–2482 (2018).
15. Yan, X. *et al.* MicroRNA-138 promotes the progression of multiple myeloma through targeting paired PAX5. *Mutat. Res.* **829**, 111869 (2024).
16. Lionetti, M. *et al.* Identification of microRNA expression patterns and definition of a microRNA/mRNA regulatory network in distinct molecular groups of multiple myeloma. *Blood* **114**, e20-26 (2009).
17. Hernández-García, S. *et al.* The kinesin spindle protein inhibitor filanesib enhances the activity of pomalidomide and dexamethasone in multiple myeloma. *Haematologica* **102**, 2113–2124 (2017).
18. Garcia-Saez, I. & Skoufias, D. A. Eg5 targeting agents: From new anti-mitotic based inhibitor discovery to cancer therapy and resistance. *Biochem. Pharmacol.* **184**, 114364 (2021).

19. Mizuhara, K. *et al.* Tumour-derived exosomes promote the induction of monocytic myeloid-derived suppressor cells from peripheral blood mononuclear cells by delivering miR-106a-5p and miR-146a-5p in multiple myeloma. *Br. J. Haematol.* **203**, 426–438 (2023).
20. Bao, Y., Wei, M. & Ji, X. MicroRNA-146b overexpression associates with deteriorated clinical characteristics, increased International Staging System stage, cacoethic chromosome abnormality, and unfavorable prognosis in multiple myeloma patients. *J. Clin. Lab. Anal.* **34**, e23168 (2020).
21. Lang, T. & Nie, Y. MiR-148a participates in the growth of RPMI8226 multiple myeloma cells by regulating CDKN1B. *Biomed. Pharmacother. Biomedecine Pharmacother.* **84**, 1967–1971 (2016).
22. Osei-Amponsa, V. *et al.* hRpn13 shapes the proteome and transcriptome through epigenetic factors HDAC8, PADI4 and transcription factor NF-κB p50. *Mol. Cell* **84**, 522-537.e8 (2024).
23. Zheng, J.-F., Guo, N.-H., Zi, F.-M. & Cheng, J. Long Noncoding RNA H19 Promotes Tumorigenesis of Multiple Myeloma by Activating BRD4 Signaling by Targeting MicroRNA 152-3p. *Mol. Cell. Biol.* **40**, e00382-19 (2020).
24. Xiang, T. *et al.* Identification of four potential predicting miRNA biomarkers for multiple myeloma from published datasets. *PeerJ* **5**, e2831 (2017).
25. Hu, S., Gao, X., Zhu, Y., Shi, F. & Huang, L. PMEPA1 Binds NEDD4L to Inhibit the Malignant Progression of Multiple Myeloma by Inactivating Wnt/β-Catenin Signaling. *Cell Biochem. Biophys.* (2025) doi:10.1007/s12013-025-01674-w.
26. Huang, X. *et al.* NEDD4L binds the proteasome and promotes autophagy and bortezomib sensitivity in multiple myeloma. *Cell Death Dis.* **13**, 197 (2022).
27. Gunnell, A., Kimber, S. T., Houlston, R. & Kaiser, M. NSD2-epigenomic reprogramming and maintenance of plasma cell phenotype in t(4;14) myeloma. *Oncotarget* **16**, 220–229 (2025).
28. Maura, F. & Bergsagel, P. L. Molecular Pathogenesis of Multiple Myeloma: Clinical Implications. *Hematol. Oncol. Clin. North Am.* **38**, 267–279 (2024).
29. Stong, N. *et al.* The location of the t(4;14) translocation breakpoint within the NSD2 gene identifies a subset of patients with high-risk NDMM. *Blood* **141**, 1574–1583 (2023).
30. Papadimitriou, M.-A. *et al.* miRNA-seq and clinical evaluation in multiple myeloma: miR-181a overexpression predicts short-term disease progression and poor post-treatment outcome. *Br. J. Cancer* **126**, 79–90 (2022).
31. Lionetti, M. *et al.* Biological and clinical relevance of miRNA expression signatures in primary plasma cell leukemia. *Clin. Cancer Res. Off. J. Am. Assoc. Cancer Res.* **19**, 3130–3142 (2013).
32. Wang, Q., Shi, Q., Wang, Z., Lu, J. & Hou, J. Integrating plasma proteomes with genome-wide association data for causal protein identification in multiple myeloma. *BMC Med.* **21**, 377 (2023).
33. Podar, K. *et al.* Vascular Endothelial Growth Factor-induced Migration of Multiple Myeloma Cells Is Associated with β1 Integrin- and Phosphatidylinositol 3-Kinase-dependent PKCα Activation*. *J. Biol. Chem.* **277**, 7875–7881 (2002).
34. Weiss, C. *et al.* Differential downregulation of telomerase activity by bortezomib in multiple myeloma cells-multiple regulatory pathways in vitro and ex vivo. *Br. J. Cancer* **107**, 1844–1852 (2012).
35. Bao, J. *et al.* N6-methyladenosine-induced miR-182-5p promotes multiple myeloma tumorigenesis by regulating CAMK2N1. *Mol. Cell. Biochem.* **479**, 3077–3089 (2024).
36. Wu, Y. *et al.* miR-182 contributes to cell adhesion-mediated drug resistance in multiple myeloma via targeting PDCD4. *Pathol. Res. Pract.* **215**, 152603 (2019).
37. Sun, Y.-Y. *et al.* Effects of miR-32 targeting PTEN on proliferation and apoptosis of myeloma cells. *Eur. Rev. Med. Pharmacol. Sci.* **24**, 3509–3516 (2020).
38. Fu, C., Wang, J., Hu, M. & Zhou, W. Circ_0005615 contributes to the progression and Bortezomib resistance of multiple myeloma by sponging miR-185-5p and upregulating IRF4. *Anticancer. Drugs* **33**, 893–902 (2022).
39. Puccio, N. *et al.* Combinatorial strategies targeting NEAT1 and AURKA as new potential therapeutic options for multiple myeloma. *Haematologica* **109**, 4040–4055 (2024).
40. Evans, R. *et al.* Aurora A kinase RNAi and small molecule inhibition of Aurora kinases with VE-465 induce apoptotic death in multiple myeloma cells. *Leuk. Lymphoma* **49**, 559–569 (2008).
41. Park, S.-S. *et al.* Predictive impact of circulating microRNA-193a-5p on early relapse after autologous stem cell transplantation in patients with multiple myeloma. *Br. J. Haematol.* **189**, 518–523 (2020).

42. Wu, Y. & Wang, H. LncRNA NEAT1 promotes dexamethasone resistance in multiple myeloma by targeting miR-193a/MCL1 pathway. *J. Biochem. Mol. Toxicol.* **32**, (2018).
43. Algarín, E. M. *et al.* Stroma-Mediated Resistance to S63845 and Venetoclax through MCL-1 and BCL-2 Expression Changes Induced by miR-193b-3p and miR-21-5p Dysregulation in Multiple Myeloma. *Cells* **10**, 559 (2021).
44. Leotta, M. *et al.* A p53-dependent tumor suppressor network is induced by selective miR-125a-5p inhibition in multiple myeloma cells. *J. Cell. Physiol.* **229**, 2106–2116 (2014).
45. Moura, S. R. *et al.* Circulating microRNAs Correlate with Multiple Myeloma and Skeletal Osteolytic Lesions. *Cancers* **13**, 5258 (2021).
46. Yu, M. *et al.* Exosomal circ-CACNG2 promotes cardiomyocyte apoptosis in multiple myeloma via modulating miR-197-3p/caspase3 axis. *Exp. Cell Res.* **417**, 113229 (2022).
47. Liu, Y. *et al.* MiR-197-3p reduces bortezomib resistance in multiple myeloma by inhibiting IL-6 expression in a MEAF6-dependent manner. *Leuk. Res.* **114**, 106785 (2022).
48. Yang, Y. *et al.* miR-137 and miR-197 Induce Apoptosis and Suppress Tumorigenicity by Targeting MCL-1 in Multiple Myeloma. *Clin. Cancer Res. Off. J. Am. Assoc. Cancer Res.* **21**, 2399–2411 (2015).
49. Gupta, N., Kumar, R. & Sharma, A. Inhibition of miR-144/199 promote myeloma pathogenesis via upregulation of versican and FAK/STAT3 signaling. *Mol. Cell. Biochem.* **476**, 2551–2559 (2021).
50. Ding, T., Deng, R. & Huang, T. Long non-coding RNA T cell factor 7 is associated with increased disease risk and poor prognosis, and promotes cell proliferation, attenuates cell apoptosis and miR-200c expression in multiple myeloma. *Oncol. Lett.* **21**, 129 (2021).
51. Handa, H., Murakami, Y., Ishihara, R., Kimura-Masuda, K. & Masuda, Y. The Role and Function of microRNA in the Pathogenesis of Multiple Myeloma. *Cancers* **11**, 1738 (2019).
52. Caracciolo, D. *et al.* miR-22 suppresses DNA ligase III addiction in multiple myeloma. *Leukemia* **33**, 487–498 (2019).
53. Łuczkowska, K. *et al.* microRNAs as the biomarkers of chemotherapy-induced peripheral neuropathy in patients with multiple myeloma. *Leuk. Lymphoma* **62**, 2768–2776 (2021).
54. Fulciniti, M. *et al.* miR-23b/SP1/c-myc forms a feed-forward loop supporting multiple myeloma cell growth. *Blood Cancer J.* **6**, e380 (2016).
55. Vlachová, M. *et al.* Involvement of Small Non-Coding RNA and Cell Antigens in Pathogenesis of Extramedullary Multiple Myeloma. *Int. J. Mol. Sci.* **23**, 14765 (2022).
56. Jia, C.-M., Tian, Y.-Y., Quan, L.-N., Jiang, L. & Liu, A.-C. miR-26b-5p suppresses proliferation and promotes apoptosis in multiple myeloma cells by targeting JAG1. *Pathol. Res. Pract.* **214**, 1388–1394 (2018).
57. Wei, X. *et al.* Multiple myeloma-derived miR-27b-3p facilitates tumour progression via promoting tumour cell proliferation and immunosuppressive microenvironment. *Clin. Transl. Med.* **13**, e1140 (2023).
58. Li, Z., Wong, K. Y., Chan, G. C.-F. & Chim, C. S. Epigenetic silencing of LPP/miR-28 in multiple myeloma. *J. Clin. Pathol.* **71**, 253–258 (2018).
59. Zhang, H. *et al.* Hypoxic Bone Marrow Stromal Cells Secrete miR-140-5p and miR-28-3p That Target SPRED1 to Confer Drug Resistance in Multiple Myeloma. *Cancer Res.* **84**, 39–55 (2024).
60. Xie, L. *et al.* Significance of a tumor microenvironment-mediated P65-miR-30a-5p-BCL2L11 amplification loop in multiple myeloma. *Exp. Cell Res.* **415**, 113113 (2022).
61. Alzrigat, M. & Jernberg-Wiklund, H. The miR-125a and miR-320c are potential tumor suppressor microRNAs epigenetically silenced by the polycomb repressive complex 2 in multiple myeloma. *RNA Dis. Houst. Tex* **4**, e1529 (2017).
62. Li, Y., Wang, L., Zhang, N. & Xu, Y. CircKCNQ5 controls proliferation, migration, invasion, apoptosis, and glycolysis of multiple myeloma cells by modulating miR-335-5p/BRD4 axis. *Histol. Histopathol.* **38**, 525–536 (2023).
63. Rio-Machin, A. *et al.* Downregulation of specific miRNAs in hyperdiploid multiple myeloma mimics the oncogenic effect of IgH translocations occurring in the non-hyperdiploid subtype. *Leukemia* **27**, 925–931 (2013).
64. Gao, L. *et al.* Metformin Inhibits Multiple Myeloma Serum-induced Endothelial Cell Thrombosis by Down-Regulating miR-532. *Ann. Vasc. Surg.* **85**, 347-357.e2 (2022).

65. Xu, K. *et al.* MicroRNA-532 exerts oncogenic functions in t(4;14) multiple myeloma by targeting CAMK2N1. *Hum. Cell* **32**, 529–539 (2019).
66. Ren, D., Cai, Y. & Xu, G. Potential of microRNA expression profile in predicting renal impairment risk in multiple myeloma patients. *Transl. Cancer Res.* **9**, 1495–1505 (2020).

Supplementary Table S2. List of 78 gene sets found by enrichment analyses of 40 targets with EnrichR; gene sets involved in Rho GTPase signaling, cell cycle, and signaling are grouped together; targes validated in gene expression database by our analyses are marked in bold.

	PATHWAYS	FDR	Genes
REACTOME	RHO GTPase Effectors	0.011	**NCKAP1L**;PRKCA;**MYH10**;MYLK;**CIT**
REACTOME	RHO GTPases Activate CIT	0.018	**MYH10**;**CIT**
REACTOME	RHO GTPases Activate PAKs	0.018	**MYH10**;MYLK
REACTOME	Signaling by Rho GTPases	0.035	**ARHGEF9**;**NCKAP1L**;PRKCA;**MYH10**;MYLK;**CIT**
REACTOME	Signaling by Rho GTPases, Miro GTPases and RHOBTB3	0.036	**ARHGEF9**;**NCKAP1L**;PRKCA;**MYH10**;MYLK;**CIT**
REACTOME	Cell Cycle	0.033	**TPX2**;IST1;**CHEK1**;**NSD2**;**HJURP**;PRKCA
HALLMARK	G2-M Checkpoint	0.013	**TPX2;CHEK1**;**NSD2**;**KIF11**
HALLMARK	Mitotic Spindle	0.045	**TPX2;KIF11**;**MYH10**
KEGG	Cellular senescence	0.018	**CHEK1**;ITPR1;ITPR2
KEGG	Apelin signaling pathway	0.015	ITPR1;ITPR2;MYLK
REACTOME	Beta-catenin Independent WNT Signaling	0.035	ITPR1;ITPR2;PRKCA
KEGG	Calcium signaling pathway	0.003	CAMK1D;ITPR1;ITPR2;PRKCA;MYLK
KEGG	cGMP-PKG signaling pathway	0.022	ITPR1;ITPR2;MYLK
REACTOME	DAG and IP3 Signaling	0.005	ITPR1;ITPR2;PRKCA
REACTOME	G Alpha (I) Signalling Events	0.044	PDE4D;ITPR1;ITPR2;PRKCA
KEGG	GnRH signaling pathway	0.008	ITPR1;ITPR2;PRKCA
REACTOME	Opioid Signalling	0.005	PDE4D;ITPR1;ITPR2;PRKCA
KEGG	Oxytocin signaling pathway	0.001	CAMK1D;ITPR1;ITPR2;PRKCA;MYLK
KEGG	Phosphatidylinositol signaling system	0.008	ITPR1;ITPR2;PRKCA
REACTOME	Platelet Activation, Signaling and Aggregation	0.033	APBB1IP;ITPR1;ITPR2;PRKCA
KEGG	Retrograde endocannabinoid signaling	0.016	ITPR1;ITPR2;PRKCA
REACTOME	Signal Transduction	0.005	SH3KBP1;PDE4D;ITPR1;ITPR2;NEDD4L;PRKCA;MYLK**;CIT**;SMPD3;APBB1IP;**KAT2B**;**ARHGEF9**;**DLG3**;**CHEK1**;**NCKAP1L;MYH10**
REACTOME	Signaling by Receptor Tyrosine Kinases	0.018	**SH3KBP1**;**CHEK1**;ITPR1;ITPR2;**NCKAP1L**;PRKCA
REACTOME	Signaling by SCF-KIT	0.039	**CHEK1**;PRKCA
REACTOME	Signaling by the B Cell Receptor (BCR)	0.048	**SH3KBP1**;ITPR1;ITPR2
REACTOME	Signaling by VEGF	0.005	ITPR1;ITPR2;**NCKAP1L**;PRKCA
KEGG	Aldosterone synthesis and secretion	0.002	CAMK1D;ITPR1;ITPR2;PRKCA
KEGG	Aldosterone-regulated sodium reabsorption	0.014	NEDD4L;PRKCA

REACTOME	Antigen Activates B Cell Receptor (BCR) Leading to Generation of Second Messengers	0.020	**SH3KBP1**;ITPR1;ITPR2
KEGG	Apoptosis	0.016	ITPR1;**EIF2AK3**;ITPR2
REACTOME	Ca2+ Pathway	0.011	ITPR1;ITPR2;PRKCA
REACTOME	Cardiac Conduction	0.035	**KAT2B**;ITPR1;ITPR2
KEGG	Cholinergic synapse	0.010	ITPR1;ITPR2;PRKCA
REACTOME	CLEC7A (Dectin-1) Induces NFAT Activation	0.009	ITPR1;ITPR2
KEGG	Cortisol synthesis and secretion	0.032	ITPR1;ITPR2
KEGG	Dopaminergic synapse	0.014	ITPR1;ITPR2;PRKCA
HALLMARK	E2F Targets	0.045	**CHEK1**;ATAD2;**CIT**
REACTOME	Effects of PIP2 Hydrolysis	0.026	ITPR1;ITPR2
REACTOME	Elevation of Cytosolic Ca2+ Levels	0.014	ITPR1;ITPR2
KEGG	Endocytosis	0.048	**SH3KBP1**;**IST1**;NEDD4L
KEGG	Gap junction	0.008	ITPR1;ITPR2;PRKCA
KEGG	Gastric acid secretion	0.001	ITPR1;ITPR2;PRKCA;MYLK
KEGG	Glioma	0.037	CAMK1D;PRKCA
REACTOME	Glucagon-like Peptide-1 (GLP1) Regulates Insulin Secretion	0.039	ITPR1;ITPR2
KEGG	Glutamatergic synapse	0.010	ITPR1;ITPR2;PRKCA
REACTOME	Glycosphingolipid Catabolism	0.036	SMPD3;ARSG
KEGG	GnRH secretion	0.005	ITPR1;ITPR2;PRKCA
REACTOME	G-protein Mediated Events	0.008	ITPR1;ITPR2;PRKCA
KEGG	Growth hormone synthesis, secretion and action	0.011	ITPR1;ITPR2;PRKCA
REACTOME	Hemostasis	0.039	APBB1IP;ITPR1;KIF13B;ITPR2;PRKCA;**KIF11**
KEGG	Human cytomegalovirus infection	0.038	ITPR1;ITPR2;PRKCA
KEGG	Human immunodeficiency virus 1 infection	0.008	**CHEK1**;ITPR1;ITPR2;PRKCA
KEGG	Inflammatory mediator regulation of TRP channels	0.008	ITPR1;ITPR2;PRKCA
REACTOME	Integration of Energy Metabolism	0.026	ITPR1;ITPR2;PRKCA
KEGG	Lipid and atherosclerosis	0.037	ITPR1;**EIF2AK3**;PRKCA
KEGG	Long-term depression	0.004	ITPR1;ITPR2;PRKCA
KEGG	Long-term potentiation	0.005	ITPR1;ITPR2;PRKCA
KEGG	Morphine addiction	0.048	PDE4D;PRKCA
REACTOME	Muscle Contraction	0.018	**KAT2B;**ITPR1;ITPR2;MYLK
KEGG	Pancreatic secretion	0.009	ITPR1;ITPR2;PRKCA
KEGG	Parathyroid hormone synthesis, secretion and action	0.002	PDE4D;ITPR1;ITPR2;PRKCA
KEGG	Parkinson disease	0.048	ITPR1;**EIF2AK3**;ITPR2
KEGG	Platelet activation	0.003	APBB1IP;ITPR1;ITPR2;MYLK

REACTOME	Platelet Calcium Homeostasis	0.026	ITPR1;ITPR2
REACTOME	PLC Beta Mediated Events	0.007	ITPR1;ITPR2;PRKCA
KEGG	Proteoglycans in cancer	0.034	ITPR1;ITPR2;PRKCA
KEGG	Regulation of actin cytoskeleton	0.037	**NCKAP1L;MYH10**;MYLK
REACTOME	Regulation of Insulin Secretion	0.015	ITPR1;ITPR2;PRKCA
KEGG	Renin secretion	0.034	ITPR1;ITPR2
KEGG	Salivary secretion	0.008	ITPR1;ITPR2;PRKCA
KEGG	Serotonergic synapse	0.010	ITPR1;ITPR2;PRKCA
KEGG	Spinocerebellar ataxia	0.016	ITPR1;ITPR2;PRKCA
KEGG	Thyroid hormone synthesis	0.006	ITPR1;ITPR2;PRKCA
KEGG	Tight junction	0.022	**DLG3**;NEDD4L;**MYH10**
REACTOME	Transmission Across Chemical Synapses	0.034	**UNC13B;ARHGEF9**;**DLG3**;PRKCA
KEGG	Vascular smooth muscle contraction	0.001	ITPR1;ITPR2;PRKCA;**MYH10**;MYLK
REACTOME	VEGFA-VEGFR2 Pathway	0.005	ITPR1;ITPR2;**NCKAP1L**;PRKCA
REACTOME	VEGFR2 Mediated Cell Proliferation	0.003	ITPR1;ITPR2;PRKCA
